# Rewetting Boreal Peatlands: Restoring Carbon Function?

**DOI:** 10.1111/gcb.70200

**Published:** 2025-04-21

**Authors:** Nigel T. Roulet, Sara Knox, Shane Regan

**Affiliations:** ^1^ Department of Geography McGill University Montreal Canada; ^2^ National Parks and Wildlife Survey Dublin Ireland

**Keywords:** boreal, carbon, peatland, restoration, rewetting

Peatlands contain some 500 ± 100 Pg of carbon (Yu 2012) stored as partially decomposed plant litter—that is, peat, even though they represent less than 4% of the terrestrial landscape (UNEP 2022). Peatlands are generally low to moderate net primary production (NPP) ecosystems but also have disproportionately low decomposition rates (i.e., ecosystem respiration—ER) than most other ecosystems due to the presence of high moisture contents inhibiting the diffusion of oxygen into the peat. This promotes more significant anaerobic decomposition, enhancing the storage of carbon in the undecomposed litter, but also leads to considerable emission of methane (CH_4_). The slight imbalance of NPP over ER for millennia has resulted in a significant global store of terrestrial carbon. On a worldwide scale, the accumulation of atmospheric CO_2_ as peat over time has resulted in net climatic cooling (Frolking and Roulet 2007). A reduction of the water stored in a peatland can increase aerobic activity, increase decomposition, and alter the imbalance to favor ER over NPP. Land use changes that involve peatland drainage typically result in peatlands becoming a source of atmospheric CO_2_ rather than a sink.

Approximately 12% of peatlands have been degraded, which usually involves some form of drainage (UNEP 2022). Degraded peatlands emit between 0.5 to 1 Pg C yr^−1^ (Leifeld and Menichetti 2018), which is equivalent to 5 to 10% of the 2014–2023 average anthropogenic emission of CO_2_ (The Global Carbon Project 2025—https://globalcarbonbudget.org/). Peatlands are deliberately drained to extract peat as a resource for fuel, which is now less common, and as a substrate for growing media. Additionally, a larger area is unintentionally drained due to land‐use changes, such as mining exploration and extraction, as well as transportation corridors. Treed peatlands are partially drained to enhance tree productivity for forestry in some northern countries (Päivänen and Hånell 2012). No matter the reason, the lowering of the water table can alter NPP, but it can also significantly increase decomposition. The Intergovernmental Panel on Climate Change (IPCC) subsidiary body on scientific and technological Advice Committee on Land Use Land‐Use Change and Forestry (LULUCF) issued a supplementary report to the IPCC Guidelines for National Inventories on Wetlands that includes the emission factors (EFs) for peatland drainage and rewetting (IPCC 2014).

The net carbon balance of a peatland comprises three components: Net ecosystem exchange of CO_2_ (NEE), CH_4_ flux, and the carbon exported as dissolved organic carbon (DOC). Undisturbed and disturbed peatlands can export a significant amount of DOC. The literature suggests that the Net Ecosystem Carbon Budget (NSECB) of undisturbed boreal peatlands is between 20 and −100 g C m^−2^ yr^−^^1^ (using the atmospheric sign convention), and the CH_4_ exchange and DOC loss reduce NEE contribution to the NECB by 20 to 40% (Yu 2012). In disturbed, drained peatlands, the NECB shifts from a carbon sink to a source, with emissions up to 10 times more than the carbon uptake in undisturbed peatlands (Petrescu et al. 2015). Identifying drained peatlands as a significant source of atmospheric carbon has led to the rewetting and restoration of drained peatlands over the last few decades (Leifeld and Menichetti 2018). The paper by Tong et al. (2025) reports the fluxes of CO_2_, CH_4_, and DOC exchange in a peatland that has been rewetted for 3 years after being partially drained for forestry purposes for over 100 years. They compare the three‐year fluxes from the rewetted peatland to the fluxes observed in the undisturbed Degero Stormyr and Hälsingfors peatlands within 14 km of each other in the same region of northern Sweden.

Tong et al. found that the rewetted peatland over the 3 years was a net source of both CO_2_ and CH_4_ but that the carbon emissions decreased in the second and third years of the study. These results are similar to those observed in the years immediately after wetting in other formerly drained peatlands, and other studies have found that it takes more than a decade for rewetted peatlands to become a sink for CO_2_ and for the NECB to become negative again (e.g., Nugent et al. 2018). The modest NEE and lower‐than‐expected CH_4_ flux result from lower biomass on the rewetted peatland. As biomass increases and the persistently wet conditions slow decomposition and reduce ER, it is reasonable to expect the NECB to switch to become negative, allowing the peatland to function as a carbon sink once again. Methane emission could rise if the density of plants CH_4_‐transporting (e.g., *Eirophoreum*) increases, along with changes in the microbial community composition, significantly affecting the overall GHG budget. In the first year, DOC loss was larger in the rewetted peatland compared to the undisturbed peatlands, but in subsequent years, the loss was similar to the other sites.

The results of Tong et al. are significant. Their estimated three‐year average EFs are less than half of what is reported by the IPCC for peatland drained for forestry (IPCC 2014). There are no direct IPCC values for comparison for boreal rewetted peatlands used for forestry, but Tong et al. values are within the range reported by Nugent et al. (2018) for the first few years of peatland restoration of peatlands used for peat extraction. However, the drop in carbon loss over the first three years, according to Tong et al., is very encouraging. As noted by Tong et al., long‐term monitoring is needed to assess whether (and when) the rewetted peatland becomes a sink for CO_2_ and whether the CH_4_ emissions stabilize around what would be expected based on the nearby undisturbed peatlands. Unfortunately, Tong et al. do not know what the fluxes were from their study peatland prior to rewetting. Without this baseline to compare the study fluxes to, a net change in EFs between the disturbed state and the rewetted state is not known. It is this change that is relevant to the change in radiative forcing of the atmosphere.

Many in the peatland science community are suggesting peatlands can play a role in nature‐based climate solutions (e.g., Girkin and Davidson 2024; Strack et al. 2022). Achieving this requires preserving carbon stored in peatlands by avoiding disturbances and converting GHG‐emitting peatlands into carbon sinks through rewetting and restoration, with long‐term restoration and protection to ensure climate benefits are realized over time. The results of Tong et al. are encouraging in this regard since the drainage in their studied peatland has been for more than a century, and this provides a basis for hope for other peatlands affected by long‐term land‐use changes, provided the critical feedback between ecology, biogeochemistry, and hydrology can be restored.

## Author Contributions

**Nigel T. Roulet:** conceptualization, writing – original draft. **Sara Knox:** conceptualization, writing – review and editing. **Shane Regan:** conceptualization, writing – review and editing.

## Conflicts of Interest

The authors declare no conficts of interest.

### Linked Article

This article is a Invited Commentary on Cheuk Hei Marcus Tong et al., https://doi.org/10.1111/gcb.70169.

## Data Availability

Data sharing not applicable to this article as no datasets were generated or analysed for the current article.
